# Obstetric and Maternal Outcomes After B-Lynch Compression Sutures: A Meta-Analysis

**DOI:** 10.7759/cureus.31306

**Published:** 2022-11-09

**Authors:** Neelam Nalini, Amit Kumar, Manoj K Prasad, Aditya V Singh, Saumya Sharma, Bijeta Singh, Triyan H Singh, Pramod Kumar, Harsh V Singh, Shreshtha Singh

**Affiliations:** 1 Obstetrics and Gynaecology, Rajendra Institute of Medical Sciences, Ranchi, IND; 2 Laboratory Medicine, Rajendra Institute of Medical Sciences, Ranchi, IND; 3 Medicine, Rajendra Institute of Medical Sciences, Ranchi, IND; 4 Medicine, Laxmi Chandravanshi Medical College, Ranchi, IND; 5 Obstetrics and Gynaecology, Medinirai Medical College, Palamu, IND; 6 Obstetrics and Gynaecology, Sri Lakshmi Narayana Institute of Medical Sciences, Puducherry, IND; 7 Biochemistry, Rajendra Institute of Medical Sciences, Ranchi, IND; 8 Ophthalmology, Sharp Sight Eye Hospital, Ranchi, IND; 9 Anaesthesia, John Hunter Hospital, New Lambton Heights, AUS

**Keywords:** postpartum hemorrhage, uterotonic drugs, pyometra, endomyometritis, b-lynch suture

## Abstract

This review article aimed to determine the obstetric and maternal outcomes after B-Lynch compression sutures to control atonic postpartum hemorrhage (PPH). This meta-analysis was performed after registering the protocol in the PROSPERO database with the registration number CRD42022355358.

Two independent reviewers systematically searched electronic databases and search engines (PubMed, Cochrane Library, and Google Scholar) to retrieve published articles from inception to July 2022. The obstetric and maternal outcomes after the B-Lynch compression suture were computed using the random-effects model in pooled proportion with a 95% confidence interval (CI). Meta-regression analysis and subgroup analysis were performed to explain any source of possible heterogeneity. Quality assessment of the included studies was done using Joanna Briggs Institute (JBI) tools which are critical appraisal tools for systematic reviews and meta-analyses.

This meta-analysis included a total of 30 studies involving 1,270 subjects. The pooled proportion of B-Lynch suture alone was 91% (95% CI = 82-97%). The combined proportion of B-Lynch suture plus another compression suture was 1% (95% CI = 0-3%), and the pooled proportion of B-Lynch suture plus vessel ligation was 3% (95% CI = 1-6%). The pooled proportions of PPH controlled and hysterectomies were 94% (95% CI = 91-97%, I^2^ = 65.3%) and 7% (95% CI = 4-10%, I^2^ = 72.13%), respectively. Therefore, B-Lynch suture (either alone or in combination with other techniques) is a simple and effective measure to control atonic PPH.

## Introduction and background

Postpartum hemorrhage (PPH) remains a significant cause of maternal mortality. PPH is the leading cause of maternal death worldwide [1,2]. Even after the availability of numerous oxytocics or uterotonic drugs such as oxytocin and ergometrine and different types of prostaglandins, various surgical methods of devascularization of the uterus, and radiological methods of embolization of uterine and internal iliac arteries, atonic PPH is a major killer of mothers after delivery in different parts of the world [3]. According to the World Health Organization (WHO), about 140,000 maternal deaths occur every year, of which 94% are in developing countries [4-8]. The B-Lynch technique to control atonic PPH was first described by Christopher B-Lynch in 1997 [9]. Since then, this innovative technique has become a popular surgical technique to control hemorrhages due to atonicity. The innovation of the B-Lynch suture was the emergence of a new era in managing life-threatening atonic PPH. Large-scale acceptance and popularity of this compression suture are because it is a simple, less time-consuming, less skill required, life-saving, fertility-preserving, and relatively safe procedure compared to vessel ligation.

B-Lynch compression suture is easy to perform and life-saving in emergencies, especially at night when intractable hemorrhage is encountered by junior surgeons such as registrars and resident doctors. However, the extreme degree of compression created by the B-Lynch suture, although life-saving, raises concerns about postoperative complications. Complications such as ischemic necrosis, infection, pyometra, Asherman syndrome, infertility, abnormal uterine bleeding, and its primary failure with resultant hysterectomy are emerging in the literature from time to time. Additionally, the literature is full of individual experiences of B-Lynch compression sutures in a variable number of cases and for a variable period. However, only a few systematic reviews [10,11] are available, and only a few randomized controlled trials (RCT) have been reported [12]. Therefore, for a surgical technique (B-Lynch suture) that is widespread and being used by every obstetrician in an emergency life-threatening situation of atonic PPH, robust and reliable data are required about its effectiveness and postoperative complications, as well as regarding its effects over subsequent pregnancies. Therefore, our team decided to perform a meta-analysis of obstetric and maternal outcomes after applying the B-Lynch compression suture.

According to the Triennial Confidential Enquiry (2000-2002), no deaths have been reported due to the B-Lynch suture. Since the invention of the B-Lynch suture in 1997, several case reports and smaller case series have been reported. However, it is difficult to arrive at any conclusion based on these case reports. We accept the hard truth that obstetricians do not always reveal a failure in case reports. The estimation of any technique’s failure rate and effectiveness is of limited value without meta-analyses, systematic reviews, and RCTs. Therefore, it is crucial to perform a meta-analysis of the B-Lynch compression suture for scientific documentation and evidence-based proof regarding its efficacy, safety, life-saving, and fertility-preserving potential.

## Review

Methodology

The meta-analysis protocol was registered in the PROSPERO database with registration number CRD42022355358. Preferred Reporting Items for Systematic Reviews and Meta-Analyses (PRISMA) guidelines for systematic review and meta-analysis 2020 edition were followed for the selection of cases/studies [13]. The PICO tool was used to formulate the research questions of this meta-analysis. The abbreviation PICO stands for P = cases of atonic PPH; I = index test: all cases in which B-Lynch suture was taken to control atonic PPH; C = comparator: B-Lynch suture versus non-surgical intervention only (we got only a few comparative studies [12]); O = outcome is determined by pooled proportion; S = study design: a meta-analysis.

Selection of Studies and Search Strategy

All published articles on B-Lynch compression suture were searched by two authors independently using Google Scholar, PubMed, and Cochrane Library. The following keywords and filters were used: B-Lynch suture and/or compression suture, and/or modified B-Lynch suture or Brace Suture. Matched references were also searched to avoid missing any articles related to the B-Lynch suture. All related articles were reviewed in detail by studying the full text for evaluation of inclusion and exclusion criteria. Articles were selected finally after quality assessment.

Inclusion Criteria

The following inclusion criteria were considered in this meta-analysis: (a) only full-text articles about B-Lynch compression suture; (b) articles about B-Lynch suture published in the English language; (c) only original articles; (d) only studies in which B-Lynch suture was applied after the failure of oxytocics/uterotonic group of drugs; and (e) articles providing sufficient information to calculate pooled proportion.

Exclusion Criteria

The following exclusion criteria were considered in this meta-analysis: (a) articles published in other languages; (b) preprint studies; (c) conference proceedings; (d) letters to the editor; (e) duplicate studies; (f) studies in which other compression sutures were used (other than B-Lynch suture) to control atonic PPH; (g) insufficient information about the outcomes (obstetric and maternal); (h) case studies; and (i) uterine balloon tamponade cases.

Quality Assessment of Studies

All studies were subjected to rigorous appraisal by two independent reviewers using Joanna Briggs Institute (JBI) tool which is a critical appraisal tool for systematic reviews and meta-analyses [14]. This tool provides a critical appraisal checklist for prevalence studies. It addresses the methodological qualities of included studies to know the possibilities of bias over three domains, namely, design, conduct, and analysis. In case of discrepancies, the third reviewer was consulted.

Extraction of Data

Two independent reviewers extracted the data from the included studies after quality assessment. The following data were extracted: author’s name, country, year of study, sample size (cases in which B-Lynch suture were taken), total number of deliveries, mean age, associated medical conditions, type of studies, type of compression suture used (B-Lynch suture alone, B-Lynch plus other compression suture, B-Lynch suture plus vessel ligation, B-Lynch suture plus uterine artery embolization (UAE)), intraoperative blood loss (in mL), pre and postoperative mean hemoglobin percentage, outcome/complication in index pregnancies (PPH controlled, hysterectomy, number of unit of blood transfusion (BT), complications due to BT, admission in intensive care unit (ICU), and other complications), suture material used, obstetric and maternal outcomes in subsequent pregnancies (rate of placenta accreta syndrome (PAS), PPH, placenta previa, preterm birth (PTB), fetal growth restriction (FGR), and others), cases of infertility including Asherman syndrome, and other gynecological problems developed subsequent to B-Lynch suture.

Statistical Analysis

The standard method recommended for meta-analysis was followed [15]. We calculated the pooled proportion with 95% CI using a random-effects model. Heterogeneity was assessed based on I^2^ value according to the Cochrane Handbook for Systematic Review of Interventions version 6.0 [16], with the following modifications: 0-30% low heterogeneity, 30-60% moderate heterogeneity, 50-90% substantial heterogeneity, and 75-100%, considerable heterogeneity. Meta-regression and subgroup analysis were performed to explain the heterogeneity. For meta-regression analysis, the following moderators/variables were considered: PPH controlled, hysterectomy performed, absorbable suture material, non-absorbable suture material, ethnicity, mean age, and type of studies. Publication bias was assessed by funnel plot and Begg’s and Egger tests. The analysis was done with the help of STATA version 13 (StataCorp., College Station, TX, USA).

Search Results of B-Lynch Suture

**Figure 1:**
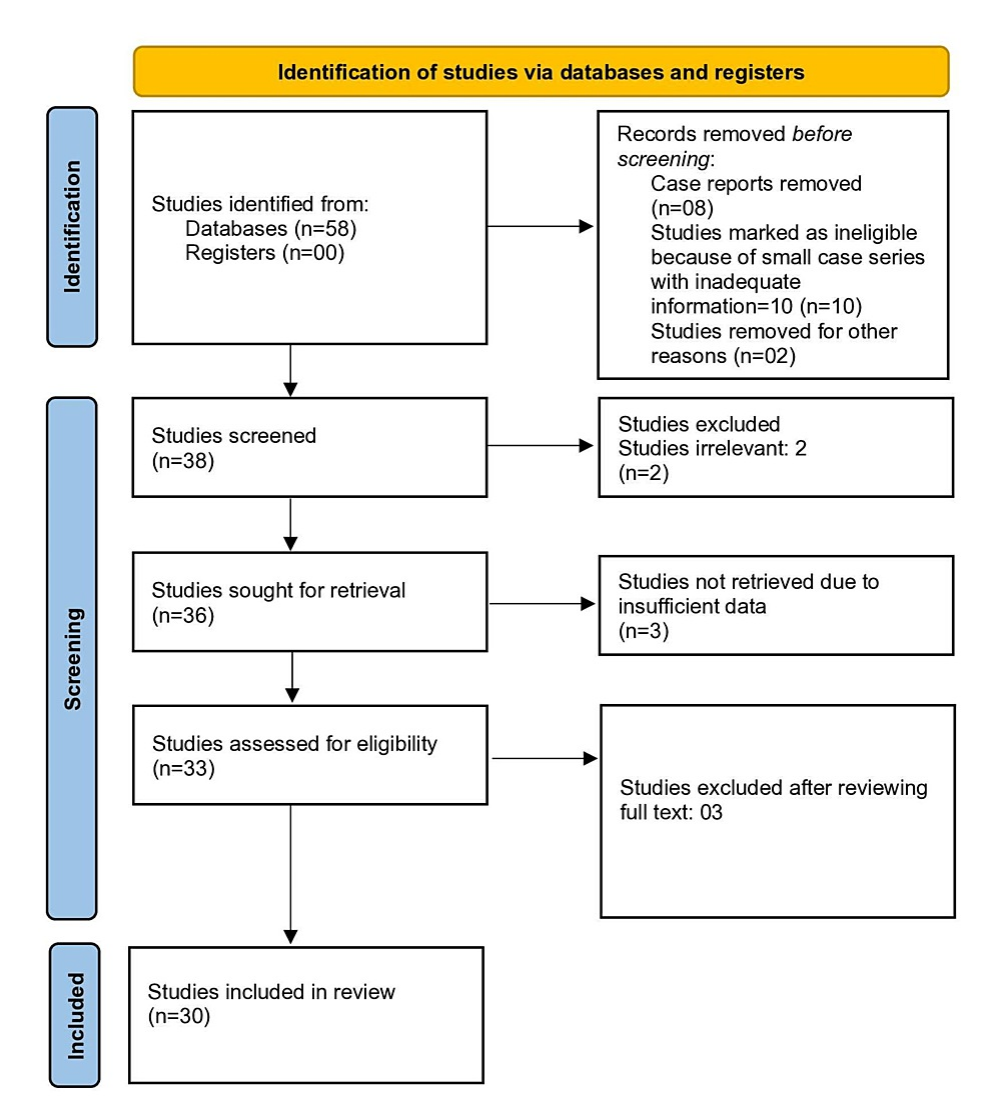
PRISMA flow diagram representing study selection and inclusion. PRISMA: Preferred Reporting Items for Systematic Reviews and Meta-Analyses

With the help of electronic databases and search engines, a total of 58 studies were identified. Out of the 58 studies, eight articles were extracted from the previous version of the review, 20 from citation searching, and 30 from searching keywords in search engines. In the initial exclusion phase, 20 studies were excluded. Out of the 20 studies, 10 were marked ineligible due to smaller case series with inadequate information, although some observational studies (either prospective or retrospective) with B-Lynch cases of less than 10 with adequate information were included. Eight case reports were removed, and the remaining two were excluded by screening title and abstract. The full text of 38 studies was reviewed, two studies were excluded due to irrelevancy, and three studies were excluded because of insufficient data, leaving 33 studies for analysis. Further, three studies were removed after reviewing the full text, leaving 30 studies for the final analysis. The PRISMA search process of included studies is shown in Figure 1.

Characteristics of Studies of B-Lynch Suture

Details of included studies of B-Lynch sutures are provided in Table 1 [12,17-45]. The total number of PPH cases in which the B-Lynch suture in these 30 studies was 1,270. Out of 30 studies, 13 were retrospective observational, 15 were prospective observational, and two were RCTs. The number of patients included in different studies ranged from six to 171. The mean age of included studies ranged from 24 to 36.3 years. In all 30 studies, the selection process of PPH cases was clearly defined.

**Table 1:** Characteristics of studies. Study coding: retrospective observational - 1, prospective observational - 2, randomized controlled trial - 3. Suture material coding: absorbable suture - 1, non-absorbable suture - 2, both absorbable and non-absorbable suture - 3. UAE: uterine artery embolization; PPH: postpartum hemorrhage; BT: blood transfusion; PTB: preterm birth; PAS: placenta accreta spectrum; FGR: fetal growth retardation

Study/Authors	Year	Country	Number of cases	Total number of deliveries	Mean age	Associated medical conditions	Type of study	B-Lynch suture alone (A)	B-Lynch suture plus other compression suture (B)	B-Lynch suture plus vessel ligation (C)	B-Lynch suture plus UAE (D)	Intraoperative blood loss (mL)	Postoperative Hb (g/dL	Outcomes/Complications in index pregnancy						Suture materials used	Obstetric and maternal outcomes in subsequent pregnancies					
														PPH controlled	Hysterectomy/Others	Number of BTs	Admission to ICU	Medical complications	Surgical complications		Rate of PAS	PPH	Placentra previa	FGR	PTB	Others
Allahdin et al. [17]	2006	Scotland	11		31.1		1	11	0	0	0	3,500		8	3	6.8		2	2	2	0	0	0	0	0	
Al Riyamiet al. [18]	2011	Canada	12	27,000	36.3		1	2	8	2	0	2,100	8.4	11	1	9				2	0	0	0		1	Myometrial necrosis in one patient, rupture uterus in one patient
Baskett et al. [19]	2007	Canada	28	31,519			2	16	12	4	1		7.5	23	5		5 pts 18%			3	0	0	0	0		
Fuglsang et al. [20]	2014	Denmark	42	53,300	32		2	42	0	0	0	2,000		41	1			8			0	0	0	0		
Pal et al. [21]	2003	India	6	4,787	23.5		2	6	0	0	0			6	0	0				1	0	0	0	0		
Holtsema et al. [22]	2003	Netherlands	7				2	0	4	3	0			7	0				1	3	0	0	0	0		
Jiang et al. [12]	2020	China	42				3	42	0	0	0	894.37		42	0						0	0	0	0		
Kaya et al. [23]	2014	Turkey	36		26.8		1	18	0	18	0	1,897		34	2	3.05	Three patient for two days	5		2	0	0	0	0		
Chai et al. [24]	2013	Hong KonG	35	26,029	28.6		1	30	0	5	0	1,650		26	9	4.02		13		1	0	0	0	0		
Koh et al. [25]	2009	Singapore	7	5,470			1	7	0	0	0	2,257		5	2	4.7		1		3	0	0	0	0		
Kong et al. [26]	2021	Hong KONG	29	20608	33.2		1	16	0	2	4	2,744		23	6	19	13 pts 44.8%				0	0	0	0		
Kwong et al. [27]	2020	Hong KONG	79	6000	32		1	29	2	35	1	2,500		69	10	2	23 pts ( 4 days stay)		3	2	0	0	0	0		
Li et al. [28]	2016	China	15		29		2	15	0	0	0	2,750		13	2	6				1	0	0	0	0		
Liu et al. [29]	2014	Singapore	23	59,665	31.5		1	23	0	1	0			20	3			1	2	1	0	0	0	0		Anatomical distortion of uterus at fundus and adhesions in one patient
Mohamed et al. [30]	2017	Egypt	171				2	171	0	0	0	1,722		168	3	2.14			23	1	0	0	0	0		
Neelam et al. [31]	2010	India	75		28	Eclampsia in nine patients	2	62	5	8	0			73	2					1	0	0	0	0		
Ouahba et al. [32]	2007	France	20				1	15	0	5	0			19	1					1	0	0	0	0		
Şahin et al. [33]	2008	Turkey	14	4000	24		2	14	0	0	0	1,696		14	0	4.2					0	0	0	0		
Sentilhes et al. [34]	2008	France	15	11,058		Auto immune thrombocytopenia in 1 pt	1	12	0	3	0	2,866		12	3	7.46		1	1		0	0	0	0		
El-Sokkary et al. [35]	2016	Egypt	160		29.6		3	160	0	0	0	3,446	8.7	144	16	3.5			14		0	0	0	0		
Songthamwat et al. [36]	2018	Thailand	57	37.843	27		1	57	0	0	0	468.4		57	0					1	0	0	0	0		
Tadakawa et al. [37]	2015	Japan	28	3976	30.8		1	26	2	0	0	2,189		26	2	4.5				1	0	0	0	0		
Zheng et al. [38]	2010	China	9	9201			1	9	0	0	0	2,894		9	0	8.7	Four patients (two-day stay)	2		1	0	0	0	0		
Sudha et al. [39]	2019	India	50		24		2	50	0	0	0	1,490	8.6	40	18											
Kalkal et al. [40]	2016	India	30		26.6		2	30	0	0	0	1,363	8.15	30	0											
Tariq et al. [41]	2011	Pakistan	60		28.8		2	60	0	0	0	1,418		57	3					1						
Qadir et al. [42]	2017	Pakistan	14				2	14	0	0	0			13	1											
Gadappa et al. [43]	2018	India	60				2	41	0	15	0			56	4				2							
Ghodake et al. [44]	2008	India	31				2	31	0	1	0			30	1				3							
Nagahama et al. [45]	2021	Brazil	104			12 patients had preeclampsia	1	104	0	0	0			99	5		16 patients									

Quality Assessment of the Included Studies

**Figure 2:**
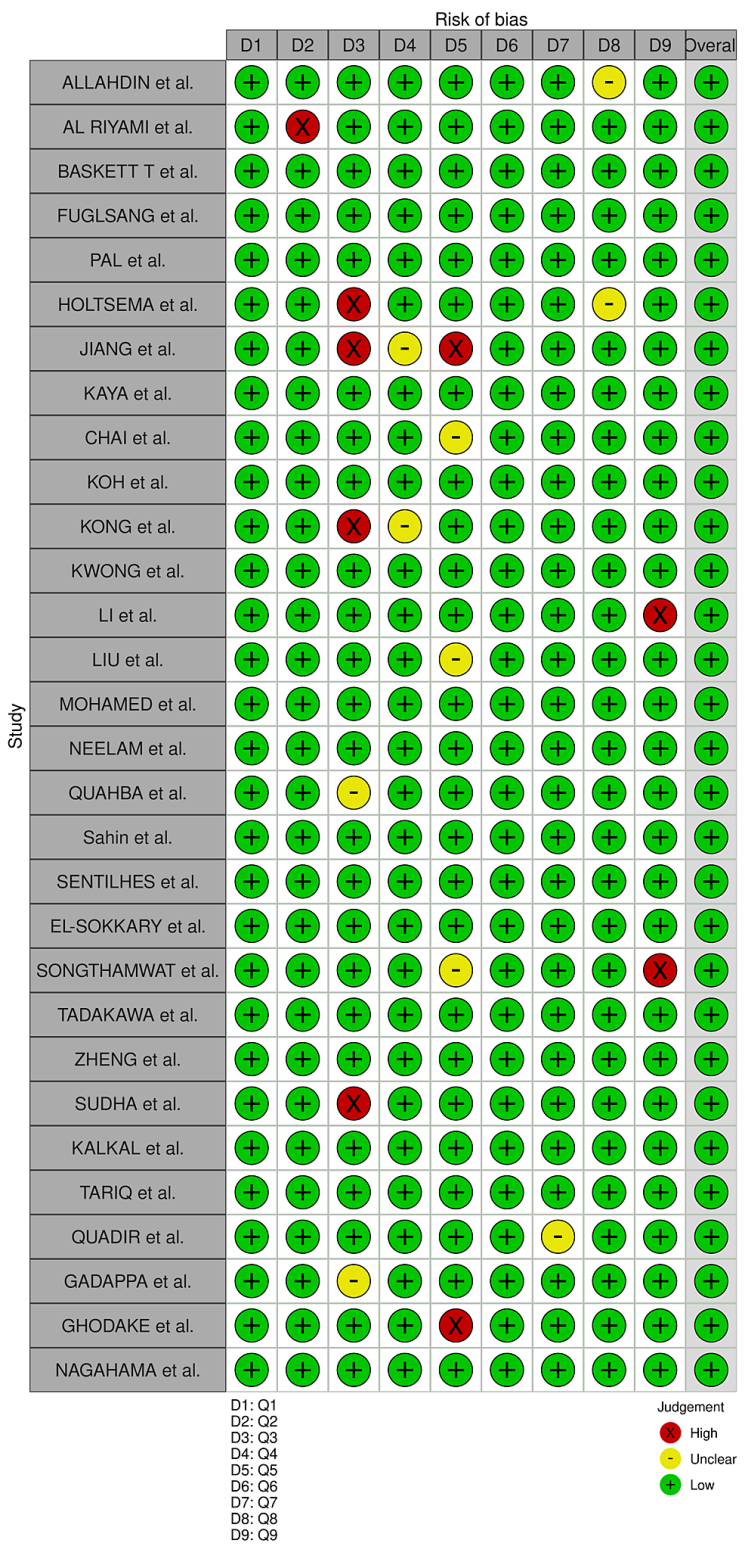
Joanna Briggs Institute tool for quality assessment. Allahdin et al. [17], Al Riyami et al. [18], Baskett et al. [19], Fuglsang et al. [20], Pal et al. [21], Holtesma et al. [22], Jiang et al. [12], Kaya et al. [23], Chai et al. [24], Koh et al. [25], Kong et al. [26], Kwong et al. [27], Li et al. [28], Liu et al. [29], Mohamed et al. [30], Neelam et al. [31], Ouahba et al. [32], Şahin et al. [33], Sentilhes et al. [34], El Sokkary et al. [35], Songthamwat et al. [36], Tadakawa et al. [37], Zheng et al. [38], Sudha et al. [39], Kalkal et al. [40], Tariq et al. [41], Quadir et al. [42], Gadappa et al. [43], Ghodake et al. [44], Nagahama et al. [45].

Figure 2 shows the detailed result of the quality assessment of included studies using the JBI tool. The result of the quality assessment demonstrated that the quality of the included studies was adequate.

Results

**Figure 3:**
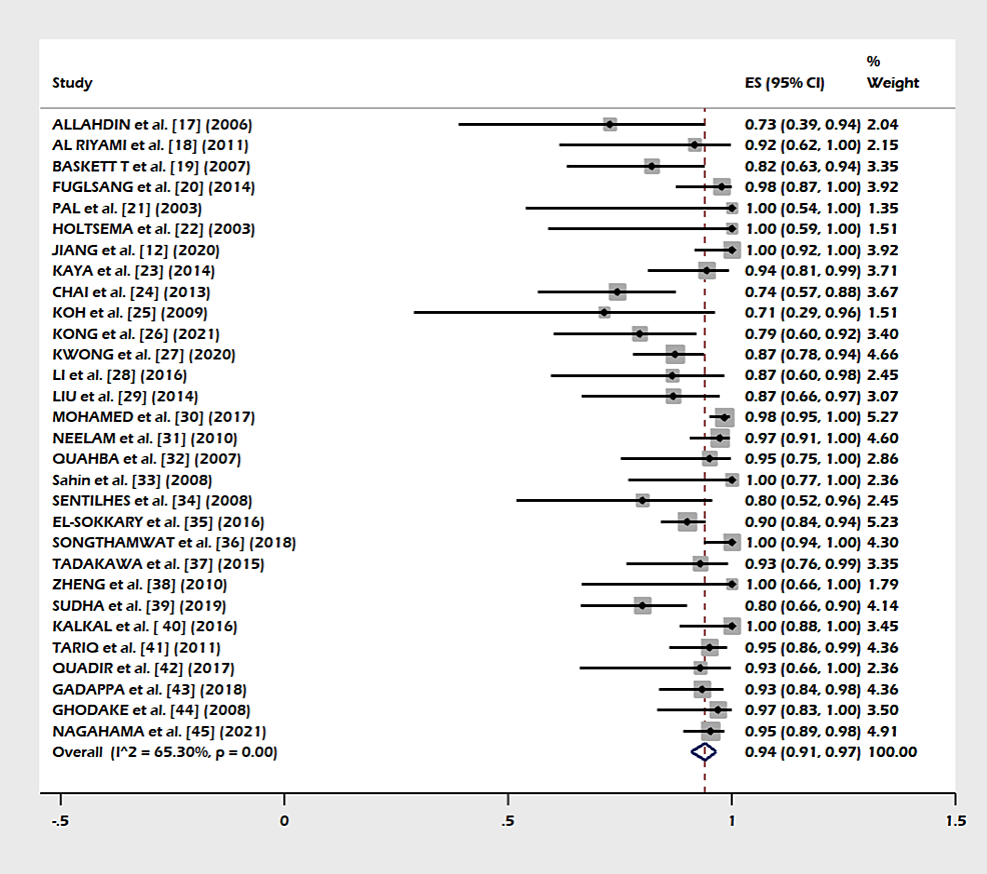
Forest plot of the pooled proportion of PPH controlled cases. Pooled proportion of PPH controlled was 94% (95% CI = 91-97%) after the B-Lynch suture. The heterogeneity was high (I^2^ = 65.3%) [12,17-45]. PPH: postpartum hemorrhage; CI: confidence interval

**Figure 4:**
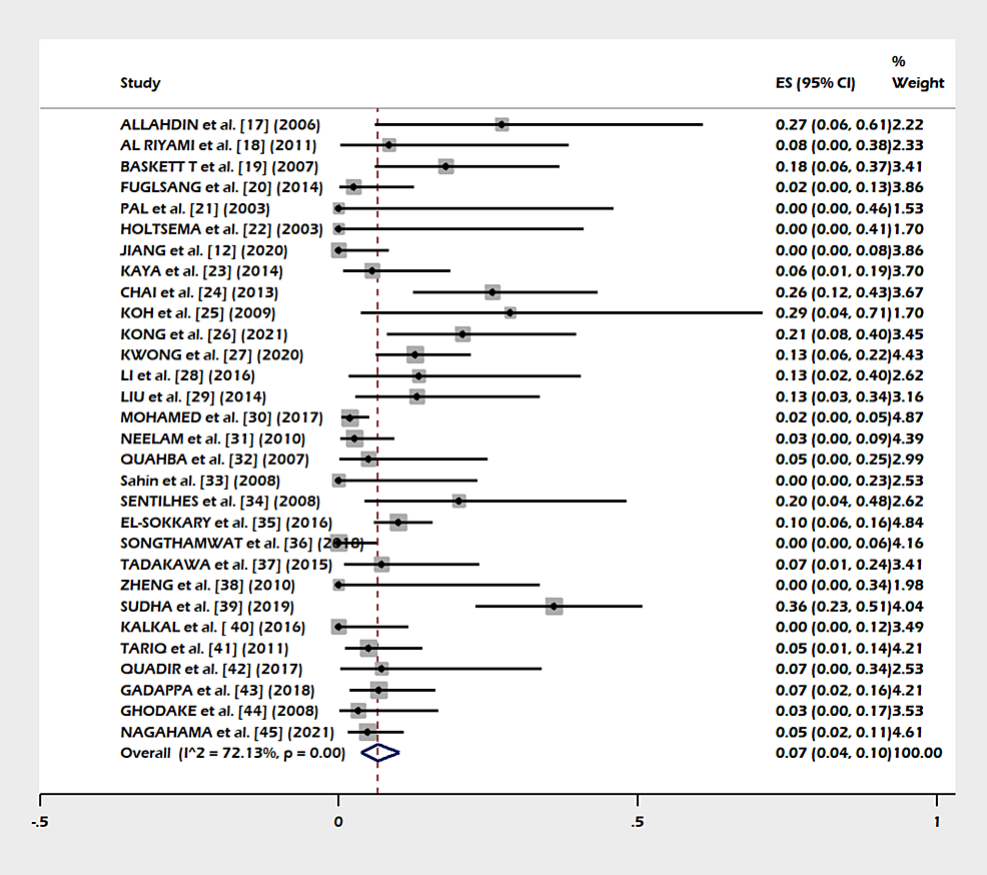
Forest plot of the pooled proportion of hysterectomy performed. The pooled proportion of hysterectomies was clinically acceptable (pooled proportion 7%, 95% CI = 4-10%). The heterogeneity was higher in this analysis (I^2^ = 72.13%) [12,17-45]. CI: confidence interval

**Figure 5:**
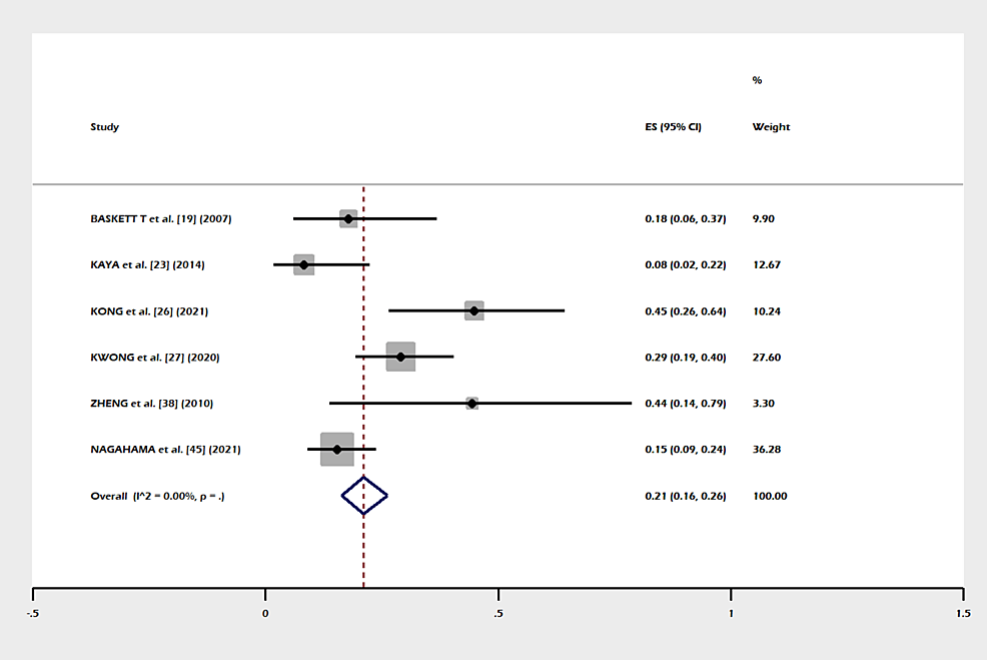
Forest plot of the pooled proportion of ICU admission. The pooled proportion of ICU admissions was 21% (95% CI = 16-26%) [19,23,26,27,38,45]. ICU: intensive care unit; CI: confidence interval

**Figure 6:**
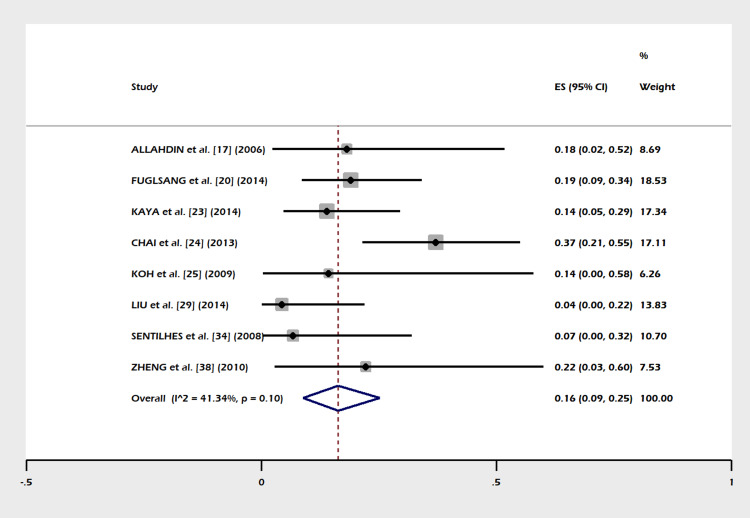
Forest plot of the pooled proportion of medical complications. The pooled proportion of medical complications associated with B-Lynch suture was 16% (95% CI = 9-25%) [17,20,23-25,29,34,38]. CI: confidence interval

**Figure 7:**
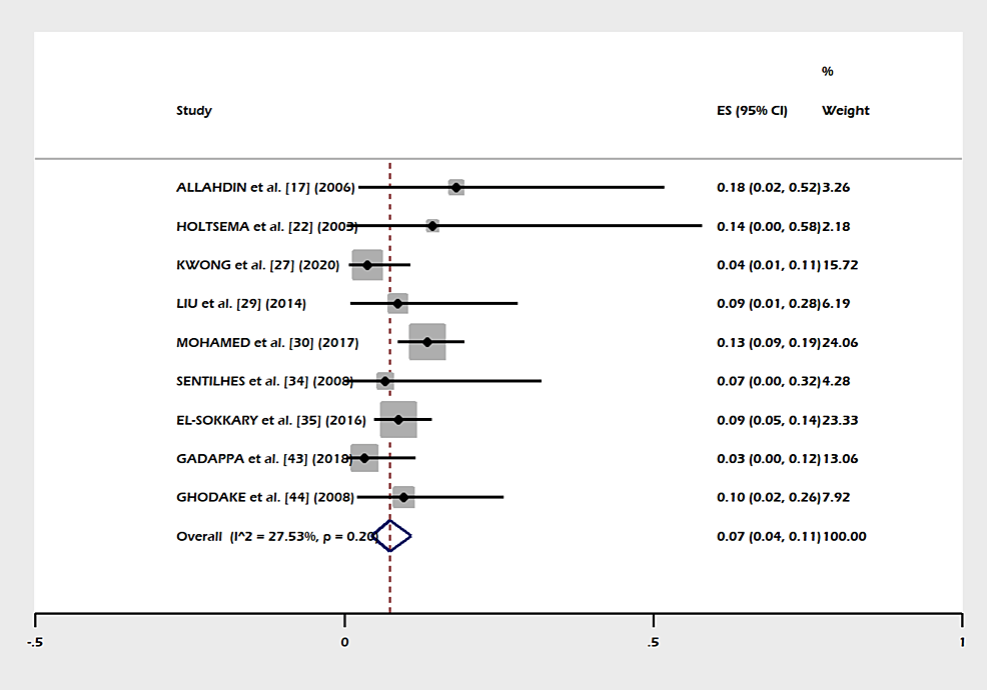
Forest plot of the pooled proportion of surgical complications. The pooled proportion of surgical complications was 7% (95% CI = 4-11%) [17,22,27,29,30,34,35,43,44]. CI: confidence interval

**Figure 8:**
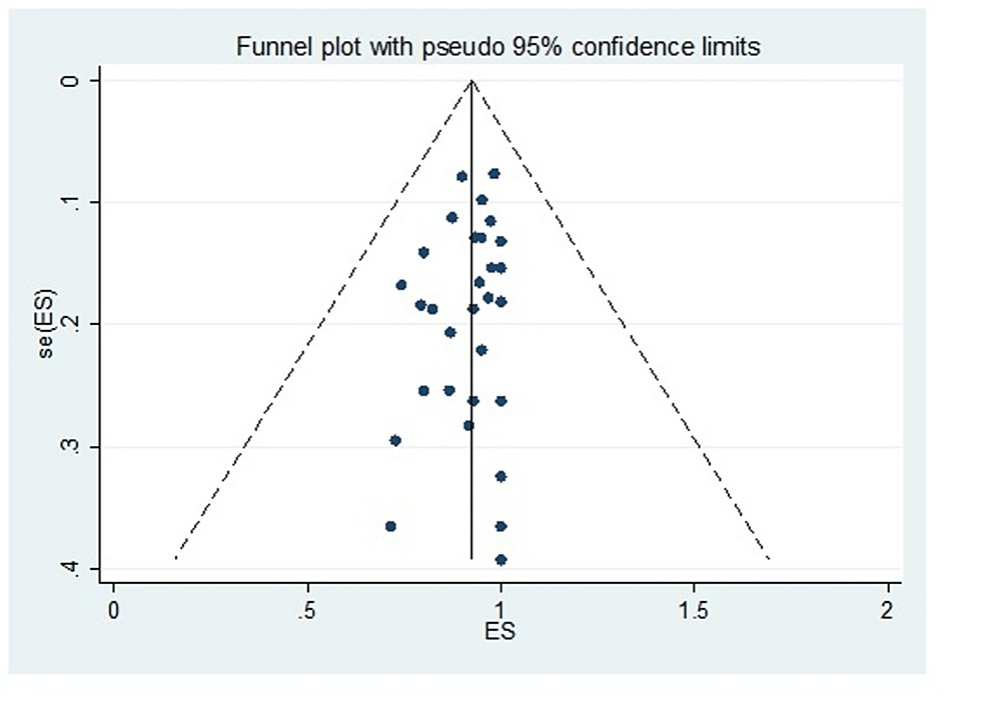
Funnel plot of PPH controlled cases. Funnel plot for assessing the publication bias for PPH controlled cases (p = 0.19). PPH: postpartum hemorrhage

**Figure 9:**
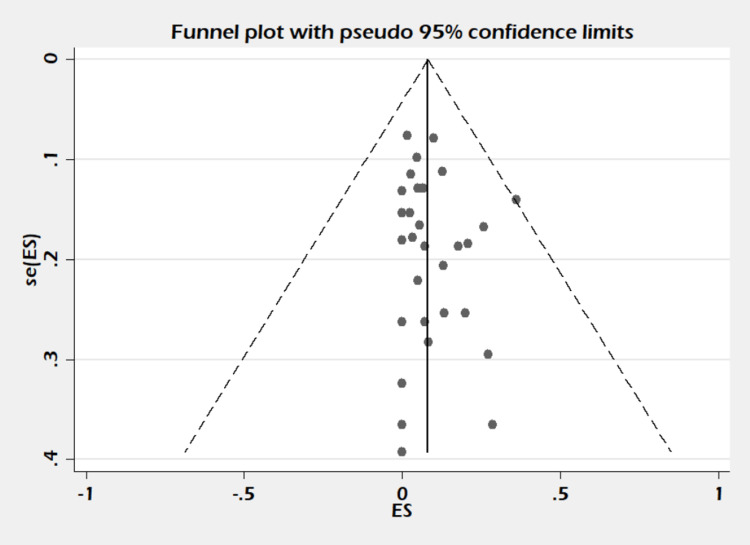
Funnel plot of hysterectomy cases performed. Funnel plot for assessing the publication bias for hysterectomies performed (p = 0.29).

A total of 30 studies including 1,270 subjects were included in this meta-analysis. The pooled proportion of B-Lynch suture alone was 91% (95% CI = 82-97%). The combined proportion of B-Lynch suture plus other compression sutures was 1% (95% CI = 0-3%). Our study observed the pooled proportion of B-Lynch suture plus vessel ligation at 3% (95% CI = 1-6%). The pooled proportion of PPH controlled was 94% (95% CI = 91-97%) (Figure 3) after the B-Lynch suture. The heterogeneity was high (I^2^ = 65.3%). The pooled proportion of hysterectomies was 7% (95% CI = 4-10%) (Figure 4). The heterogeneity was higher in this analysis (I^2 ^= 72.13%). The pooled proportion of ICU admissions was 21% (95% CI = 16-26%) (Figure 5) after the B-Lynch suture. The pooled proportion of medical complications (pulmonary edema, postoperative endomyometritis, postpartum cardiomyopathy, pulmonary embolism, pyelonephritis, disseminated intravascular coagulation (DIC)) associated with B-Lynch suture was 16% (95% CI = 9-25%) (Figure 6), while the pooled proportion of surgical complications (relaparotomy, hematometra, pyometra, retained products of conception, bladder injury, wound hematoma) was 7% (95% CI = 4-11%) (Figure 7). We did not observe significant publication bias in the pooled proportion of PPH controlled and hysterectomy done (p = 0.19 and p = 0.29, respectively), which is also visible in the funnel plots (Figures 8, 9).

Meta-Regression/Subgroup Analysis

In the subgroup analysis, we found that 96% of PPH was controlled in the group that used absorbable suture materials (95% CI = 91-99%), followed by the group that used non-absorbable suture materials at 90% (95% CI = 83-95%) and the group that used both absorbable and non-absorbable suture materials at 87% (95% CI = 69-98%). In the subgroup analysis, we observed that the pooled proportion of hysterectomies done in the absorbable suture material used group was 4% (95% CI = 1-9%), followed by the non-absorbable group at 10% (95% CI = 5-17%) and both suture material groups at 13% (95% CI = 2-31%). Subgroup analysis according to ethnicity showed that the pooled proportion of PPH controlled in the Caucasian group was 93% (95% CI = 86-98%), and in the Asian group, the pooled proportion of PPH controlled was 94% (95% CI = 91-97%). In the Caucasian group, the pooled proportion of hysterectomies done was 7% (95% CI = 2-14%), whereas in the Asian group, it was 6% (95% CI = 3-10%). We found no significant influence of study type, mean age, ethnicity, or suture material used on the pooled proportion for PPH controlled (p = 0.64, 0.72, 0.93, and 0.50, respectively) and for hysterectomy (p = 0.69, 0.82, 0.98, and 0.50, respectively).

Discussion

Widespread acceptance of B-Lynch suture is because it is easy to learn, easy to gain expertise in, and competency and proficiency levels can be achieved fast. Moreover, there is no risk of injury to the ureter and major vessels. There are several surgical techniques to control PPH. However, it requires a considerable degree of experience and surgical expertise. After the innovation of the B-Lynch compression suture, several types of compression sutures were reported in the literature such as the Cho-Square compression suture, Hayman technique, Pereira compression suture, Ouahba technique of compression suture, and Hackethan technique [10]. However, these compression sutures are not without complications and are not uniformly safe [46]. The relative safety of the B-Lynch suture is because it does not sew the anterior and posterior uterine walls together which is in contrast to other compression sutures [47]. In another type of uterine compression sutures where the anterior and posterior walls of the uterus are sewed together, complications such as pyometra, hematometra, infection, ischemic necrosis, Asherman syndrome, and infertility are relatively high.

This meta-analysis noted few complications in the index pregnancy (Figures 5-7), good control of PPH (Figure 3), and a small failure rate in the form of hysterectomy (Figure 4) but almost (except a few) no complications in a subsequent pregnancy (Table 1).

Success/Failure Rate

In our meta-analysis PPH, controlled cases are shown in Figure 3). The failure rate in the form of a hysterectomy is shown in Figure 4). A success rate of 91.7% in controlling PPH (atonic) is reported in other systematic reviews [10,46,47,48] in the literature.

Complications in Index Pregnancy

Different types of postoperative complications were noted, including both medical and surgical complications (Figures 6, 7). One of the complications encountered was postoperative endometritis. This B-Lynch compression suture acts by creating compression over both the anterior and posterior uterine walls. The degree of compression remains so high that there is no space between the anterior and posterior uterine walls and the endometrial cavity is completely obliterated. Hence, all uterine venous channels are compressed and closed which results in the stoppage of bleeding. Unlike stepwise devascularization or vessel ligation, it does not stop the blood supply to the uterus. Hence, complications such as postoperative ischemia or ischemic necrosis, which is comparatively higher with vessel ligation procedures or with UAE are less frequent with B-Lynch sutures. These types of complications have been noted more in cases where the B-Lynch suture is combined with vessel ligation or UAE. While tightening the suture in the B-lynch procedure, it should be tight enough so that the uterine cavity remains completely obliterated. Loose B-Lynch sutures do not provide the desired result and lead to complications such as hematometra and superadded infection converted to endometritis and pyometra. Too tight sutures in the B-Lynch procedure probably result in ischemic necrosis, Asherman syndrome, and infertility. However, in our meta-analysis, only very small associations were noted (Figures 6, 7, Table 1). The above two complications might be considered as two ends of the spectrum. This can also be explained by the type of suture used in the B-Lynch suture. The probable explanation is that with the use of absorbable sutures, such as chromic catgut, enough tightening is not possible (because it often breaks down) which might be associated with pockets of potential space in the uterine cavity. Blood collects here and resulted in hematometra and later on endomyometrits/pyometra. On the other hand, in cases where non-absorbable sutures or delayed absorbable sutures are used, there might be the possibility of the highest degree of tightening, and the resultant extreme degree of compression might give rise to more incidences of ischemic necrosis of the uterine wall, Asherman syndrome, and infertility. However, the uterus is a dynamic organ, and in the puerperium, it undergoes continuous involution. Therefore, this daily decrease in the dimension of the uterus results in the loosening of the suture after 24 hours, with the possibility of ischemic necrosis being remote. This is also evident in our meta-analysis.

A systematic review of the needle and suture types for uterine compression sutures was reported by Matsuzaki et al. in 2019 [49]. However, because of the heterogeneity of cases included in their studies, it was difficult to conclude which suture should be preferred for uterine compression. Hence, to get optimum outcomes, more studies and meta-analyses are required to know about the type of suture perfect for uterine compression suture (UCS).

In our meta-analysis, DIC was an important postoperative complication (Figure 6). DIC appears to be the result of massive hemorrhage and should not be considered a complication of B-Lynch suture. Hence, whenever medical interventions in the form of oxytocic fail, without unnecessary wastage of time, another method in the form of compression suture should be used. Because once a massive hemorrhage has already occurred, it leads to disturbance of the clotting cascade, washing out of the clotting factors, and ultimately DIC. Therefore, this time gap between the start of atonic PPH and the decision of using a B-Lynch suture is very important. So, every hospital must have its sequential protocol for the management of atonic PPH.

In one of the studies, bladder injury was present in a significant number of cases [30]. The probable explanation might be a technical error (as B-Lynch suture is taken after separating and pushing down the utero-vesical fold of the peritoneum which keeps the urinary bladder out of danger).

A combination of B-Lynch suture plus other compression sutures and/or vessel ligation was used more commonly in placenta previa cases. As the placenta is implanted in the lower uterine segment, an additional isthmic-cervical apposition suture was also used in these cases to produce a compression effect over the lower uterine segment. An adequate compression effect is not created by the B-Lynch suture in the lower uterine segment.

Complications in Subsequent Pregnancy

In our meta-analysis, no significant obstetrical and maternal complications were observed in subsequent pregnancies. Anatomical distortion of the uterus and rupture uterus was observed in sporadic cases only (Table 1). Moreover, no significant gynecological effect was observed.

In addition to different types of compression sutures, UAE has also become popular as a fertility-preserving, non-surgical modality to control PPH. However, UAE is not possible in a resource-poor setup. Because of the simplicity of the B-Lynch suture, it has also become popular in developing countries with poor resource setups.

Placenta accreta syndrome (PAS) is always notorious for massive PPH and high maternal mortality. PAS is associated with significant blood loss approximately (3,000 mL) and the rate of hysterectomy is also high [50-54]. Jauniax et al. [52] in their meta-analysis and systematic review of PAS reported the same findings.

Recently, Matsuzaki et al. [55] in another meta-analysis and systematic review observed a significant association between PPH in a subsequent pregnancy, PAS, rate of hysterotomy, and history of prior UAE in a previous pregnancy (OR = 42.38, 95% CI = 11.00-163.25; heterogeneity: I^2 ^= 73, p = 0.03).

In our meta-analysis, we did not observe an association between B-Lynch suture and PAS in a subsequent pregnancy. Moreover, no cases of preterm birth or fetal growth restriction were observed. However, long-term follow-up in a subsequent pregnancy was not available in many of the studies which was a major limitation of our meta-analysis.

Strength of the study

This is the first meta-analysis of the obstetric and maternal outcomes of B-Lynch sutures. The main strength of the study is that we strictly followed the guidelines and standard methodology of meta-analysis. For the selection of studies, PRISMA guidelines were followed. Quality assessment of included studies was done strictly using JBI tools. A potential source of heterogeneity was explored by meta-regression and subgroup analysis.

Limitations of the study

We only included studies published in the English language. Hence, we could not avoid language biases in study selection. Because many of the included studies were retrospective, bias was not measured. We found only two comparator studies. A comparator group study was not possible in the majority of studies. Because the first step to controlling atonic PPH is the use of oxytocics/uterotonics that has universal acceptance. The decision of compression suture (B-Lynch and/or other measures) is taken only after the failure of oxytocics. Hence, the action plan to control atonic PPH is sequential (one after another). In many of the studies, the sample size was limited. Therefore, there is a possibility of type II errors. Long-term follow-up following B-Lynch suture was not available in many studies. Many subjects denied further pregnancy because of the experience of PPH, admission to the ICU, and complications related to BT. Therefore, robust data on the effect on subsequent pregnancy could not be drawn. Moreover, it was difficult to extract the cause of the failure to control bleeding (PPH), whether it was the failure of surgical technique (B-Lynch suture) or the associated systemic condition of the mother. In most studies, the systemic condition of the mother (hypertensive disorder/clotting disorder/systemic lupus erythematosus/heart disease on an anticoagulant) was not mentioned. Failure may be due to a late decision to take a B-Lynch suture when already massive hemorrhage had occurred with the patient in severe shock with the vicious cycle of DIC. However, this crucial time interval between the onset of atonic PPH and the intervention decision was not mentioned in almost all studies. In most studies, pre and postoperative hemoglobin percentages were not mentioned, making it difficult to know the efficacy of the technique.

## Conclusions

The B-Lynch suture is a simple and effective technique to control atonic PPH. Its fertility-preserving and life-saving potential is high, and it can be used in low-resource setups. It should be included in the training curriculum of residents and registrars and should be incorporated into the protocol of PPH control of every hospital. However, more RCTs and meta-analyses are required in this field, especially regarding the type of suture used. Further studies with background-matched patient cohorts are required. Moreover, there is a need for long-term follow-up of cases of B-Lynch sutures to know their effect on subsequent pregnancies and their gynecological outcomes.
